# HIV-1 Infection in Cyprus, the Eastern Mediterranean European Frontier: A Densely Sampled Transmission Dynamics Analysis from 1986 to 2012

**DOI:** 10.1038/s41598-017-19080-5

**Published:** 2018-01-26

**Authors:** Andrea-Clemencia Pineda-Peña, Kristof Theys, Dora C. Stylianou, Ioannis Demetriades, SPREAD/ESAR Program, Elisabeth Puchhammer, Anne-Mieke Vandamme, Ivailo Aleksiev, Snjezana Zidovec Lepej, Marek Linka, Jannik Fonager, Kirsi Liitsola, Rolf Kaiser, Osamah Hamouda, Dimitrios Paraskevis, Suzie Coughlan, Zehava Grossman, Orna Mor, Maurizio Zazzi, Algirdas Griskevicius, Vilnele Lipnickiene, Carole Devaux, Charles Boucher, Marije Hofstra, Annemarie Wensing, Anne-Marte Bakken-Kran, Andrzej Horban, Ricardo Camacho, Simona Paraschiv, Dan Otelea, Maja Stanojevic, Danika Stanekova, Mario Poljak, Federico Garcia, Roger Paredes, Jan Albert, Ana B. Abecasis, Leondios G. Kostrikis

**Affiliations:** 1 Global Health and Tropical Medicine-GHTM, Institute for Hygiene and Tropical Medicine, Universidade NOVA de Lisboa, UNL, Lisbon, Portugal; 2 Molecular Biology and Immunology Department, Fundación Instituto de Inmunología de Colombia (FIDIC); Basic Sciences Department, Universidad del Rosario, Bogotá, Colombia; 3 Katholieke University of Leuven, Department of Microbiology and Immunology, Rega Institute for Medical Research, Clinical and Epidemiological Virology, B-3000 Leuven, Belgium; 4 Laboratory of Biotechnology and Molecular Virology, Department of Biological Sciences, University of Cyprus, Nicosia, Cyprus; 5 AIDS Clinic, Larnaca National Hospital, Larnaca, Cyprus; 6 Medical University Vienna, Vienna, Austria; 7 National Center of Infectious and Parasitic Diseases, Sofia, Bulgaria; 8 University Hospital for Infectious Diseases “Dr. Fran Mihaljevic”, Zagreb, Croatia; 9 National Reference Laboratory for HIV/AIDS, National Institute of Public Health, Prague, Czech Republic; 10 Statens Serum Institute, Copenhagen, Denmark; 11 Department of Health Security, National Institute for Health and Welfare, Helsinki, Finland; 12 Institute of Virology, University of Cologne, Cologne, Germany; 13 Robert Koch Institute, Berlin, Germany; 14 National Retrovirus Reference Center, University of Athens, Athens, Greece; 15 University College Dublin, Dublin, Ireland; 16 Tel Aviv University, Tel Aviv, Israel; 17 National HIV Reference Laboratory, Public Health Services, Chaim Sheba Medical Center, Tel-Hashomer, Israel; 18 University of Siena, Siena, Italy; 19 Lithuanian AIDS Center, Vilnius, Lithuania; 20 Department of Infection and Immunity, Laboratory of Retrovirology, Luxembourg Institute of Health, Luxembourg, Luxembourg; 21 Erasmus MC, University Medical Center, Rotterdam, The Netherlands; 22 University Medical Center Utrecht, Virology, Utrecht, The Netherlands; 23 University of Oslo, Oslo, Norway; 24 Hospital of Infectious Diseases, Warsaw, Poland; 25 Molecular Diagnostics Laboratory, National Institute for Infectious Diseases, Bucharest, Romania; 26 University of Belgrade Faculty of Medicine, Belgrade, Serbia; 27 Slovak Medical University, Bratislava, Slovakia; 28 Slovenian HIV/AIDS Reference Centre, University of Ljubljana, Faculty of Medicine, Ljubljana, Slovenia; 29 Department of Clinical Microbiology, Hospital Universitario San Cecilio de Granada,Instituto de Investigacion Biosanitaria(IBIS), Granada, Spain; 30 IrsiCaixa AIDS Research Institute, Badalona, Spain; 31 Karolinska Institute, Solna, Sweden; 32 Karolinska University Hospital, Stockholm, Sweden

## Abstract

Since HIV-1 treatment is increasingly considered an effective preventionstrategy, it is important to study local HIV-1 epidemics to formulate tailored preventionpolicies. The prevalence of HIV-1 in Cyprus was historically low until 2005. To investigatethe shift in epidemiological trends, we studied the transmission dynamics of HIV-1 in Cyprususing a densely sampled Cypriot HIV-1 transmission cohort that included 85 percent ofHIV-1-infected individuals linked to clinical care between 1986 and 2012 based on detailedclinical, epidemiological, behavioral and HIV-1 genetic information. Subtyping andtransmission cluster reconstruction were performed using maximum likelihood and Bayesianmethods, and the transmission chain network was linked to the clinical, epidemiological andbehavioral data. The results reveal that for the main HIV-1 subtype A1 and B sub-epidemics,young and drug-naïve HIV-1-infected individuals in Cyprus are driving the dynamics of thelocal HIV-1 epidemic. The results of this study provide a better understanding of thedynamics of the HIV-1 infection in Cyprus, which may impact the development of preventionstrategies. Furthermore, this methodology for analyzing densely sampled transmissiondynamics is applicable to other geographic regions to implement effective HIV-1 preventionstrategies in local settings.

## Introduction

Approximately 37 million people were living with human immunodeficiency virus (HIV) in 2016, with almost 2 million new infections and 1 million AIDS-related deaths^1^. Recently, the number of newly diagnosed HIV infections has increased in several European countries. The HIV prevalence in Cyprus was among the lowest in Europe until 2005^2^, with an HIV-1 infection rate of 5.6 per 100,000 population in 2005. However, since 2005, the number of newly diagnosed individuals has increased, and the rate of HIV-1 infections was 6.5 per 100,000 in 2014^3^. It remains unclear whether this finding shows a genuine increase in the incidence or whether it is due to other factors, such as more efficient diagnostic capabilities^2^. Understanding the reason for this increase and identifying those within the population who are responsible for and at risk of HIV-1 transmission is pivotal in this setting.

The Cypriot epidemic is an extremely interesting model for studying HIV-1 transmission for several reasons. First, a complete and densely sampled dataset containing socio-demographic, clinical, virological and behavioral data has been continuously maintained over the years. For example, until 2009, approximately 88% of the HIV-1 epidemic in Cyprus had been included in this collection^4,5^. According to the most recent molecular epidemiology study in Cyprus, the most frequent circulating HIV-1 subtypes were A (19%) and B (49%)^4^. This dataset is ideal for determining the characteristics of the population involved in the transmission of an HIV-1 infection or those who are at risk of infection given its up-to-date information. Second, this epidemic includes two different subtypes (A and B) that are circulating together^4,5^ on the island.

The resolution of methodologies used to infer transmission dynamics has significantly improved in recent years^6,7^. Although transmission clusters (TCs) have been identified through various mechanisms, phylogenetic cluster analyses using molecular surveillance and clinical data represent the most common and widely applicable approach used to reconstruct transmission histories and identify epidemiological linkage between HIV-1-infected individuals^8,9^. Therefore, we used up-to-date phylogenetic analysis methods and transmission chain reconstruction to analyze the HIV-1 epidemic in Cyprus in depth and describe and characterize HIV-1 transmission in this country. We aimed to identify the factors that led to the increase in HIV-1 incidence in Cyprus and the self-sustainment of the concurrent HIV-1 subtype B and non-B subtype sub-epidemics.

## Methods

### Study population

HIV-1 nucleotide sequences and clinical and epidemiological information were retrieved from 336 HIV-1-infected patients from Cyprus as part of four previous molecular epidemiological studies investigating HIV-1 infection in Cyprus. The samples were collected in accordance with relevant guidelines and regulations of the National Bioethics Committee in Cyprus^4,5,10,11^. Specifically, in the first study, newly diagnosed untreated HIV-1 patients, representing 72% of the total number of newly diagnosed and drug-naïve patients during the period from 2003 to 2006, were recruited from the AIDS Clinic of Larnaca General Hospital in Cyprus^11^. In the second study, HIV-1-infected individuals were recruited during the period from 1986 to 2006, representing 38% of the known infected population in Cyprus^5^. In the third study, HIV-1-infected individuals were recruited during the period from 2007 to 2009, representing 88% of the known-living HIV-1-infected population, with 53 newly diagnosed therapy-naive patients and 21 chronic patients according to the European HIV Resistance guidelines^4^. Furthermore, the group of study subjects included newly diagnosed and chronic drug-naïve HIV-1-infected patients during the period from 2010 to 2012 (L.G. Kostrikis *et al*., manuscript in preparation for publication) and chronic patients during the period from 1986 to 2012. All experimental protocols were approved by the National Bioethics Committee in Cyprus^4,5,10,11^. The present molecular epidemiology study compiles data from the aforementioned molecular epidemiology studies in Cyprus in accordance with relevant guidelines and regulations of the National Bioethics Committee in Cyprus and the Office of the Commissioner for Personal Data Protection in Cyprus, and in accordance with the written consent of all participating study subjects. To determine whether this cohort is representative of the total cumulative cases reported in Cyprus, data from our cohort (Table 1) were compared with the available data collected from the European Centre for Disease Prevention and Control (ECDC)^2,12^.

**Table 1:** Characteristics of the Cypriot cohort and the transmission clusters. The Cypriot cohort, the population in the TCs and the population in clusters are shown. Abbreviations: HCV: Hepatitis C virus; HBsAg: surface antigen of the hepatitis B virus; IQR: interquartile range; n: sample; MSM: men who have sex with men; p: p-value for the univariate analysis; SD: standard deviation; TCs: transmission clusters.

Variable	Total cohort		Cohort in TCs		Cohort out of TCs		p-value	Cohort in clusters ≥3		Cohort out of clusters ≥3		p-value
	n = 336	%	n = 163	%	n = 144	%		n = 96	%	n = 211	%	
**Gender** ^**2**^												
Male	256	77.3	122	76.7	114	79.7	0.578	77	81.1	159	76.8	0.456
Female	75	22.7	37	23.3	29	20.3		18	19.0	48	23.2	
Unknown*	5	1.5	4	2.5	1	0.7		1	1.0	4	1.9	
Age [SD]^**3**^	38.42	[11.97]	37.97	[12.75]	39.1	[11.36]	0.418	34.6	[10.03]	40.27	[12.55]	0
**Age categories** ^**1**^												
<25	27	8.2	13	8.3	12	8.4	0.569	9	9.7	16	7.8	0.008
25–34	110	33.6	54	34.6	46	32.2		37	39.8	63	30.6	
35–44	92	28.1	47	30.1	36	25.2		32	34.4	51	24.8	
45–55	74	22.6	30	19.2	39	27.3		13	14.0	56	27.2	
>55	24	7.3	12	7.7	10	7.0		2	2.2	20	9.7	
Unknown*	9	2.7	7	4.3	1	0.7		3	3.1	5	2.4	
**Risk Factor** ^**1**^												
MSM	164	52.1	85	55.9	71	51.1	0.883	60	65.2	96	51.3	0.019
Heterosexual	129	41.0	60	39.5	56	40.3		29	31.5	87	46.5	
Other	22	7.0	7	4.6	12	8.6		3	3.3	4	2.1	
Unknown*	21	6.3	11	6.8	5	3.5		4	4.2	24	11.4	
**Area of Residence** ^**2**^												
Urban	217	66.4	107	67.7	94	66.2	0.807	61	64.9	140	68.0	0.599
Rural	110	33.6	51	32.3	48	33.8		33	35.1	66	32.0	
Unknown*	9	2.7	5	3.1	2	1.4		2	2.1	5	2.4	
**City of Residence** ^**1**^												
Nicosia	131	40.2	61	38.6	58	40.9	0.286	39	41.5	80	38.8	0.953
Limassol	103	31.6	57	36.1	40	28.2		30	31.9	67	32.5	
Larnaca	60	18.4	24	15.2	31	21.8		17	18.1	38	18.5	
Other	32	9.8	16	10.1	13	9.2		8	8.5	21	10.2	
Unknown*	10	3.0	6	3.1	2	1.4		2	2.1	5	2.4	
**Country of origin** ^**2**^												
Cyprus	233	70.6	116	73.0	100	70.6	0.803	71	70.6	145	68.7	0.419
Other	97	29.4	43	27.0	42	29.4		24	29.4	66	31.3	
Unknown*	6	1.8	4	2.5	2	1.4		1	1.0	0	0	
**Region of origin** ^**1**^												
Cyprus	233	70.6	116	73.0	100	70.4	0.213	71	74.7	145	68.7	0.014
Western Europe	2	0.6	1	0.6	0	0.0		1	1.1	0	0.0	
Eastern Europe	27	8.2	12	7.5	13	9.2		7	7.4	18	8.5	
Northern Europe	11	3.3	8	5.0	3	2.1		6	6.3	5	2.4	
Southern Europe	11	3.3	8	5.0	2	1.4		6	6.3	4	1.9	
Western Asia	7	2.1	1	0.6	6	4.2		0	0.0	7	3.3	
Eastern Africa	4	1.2	2	1.3	2	1.4		1	1.1	3	1.4	
Middle Africa	16	4.8	5	3.1	6	4.2		0	0.0	11	5.2	
Other	19	5.8	6	3.8	10	7.0		3	3.2	18	8.5	
Unknown*	6	1.8	4	2.5	2	1.4		1	1.0	0	0	
**Country of Infection** ^**2**^												
Cyprus	132	51.0	76	61.8	51	42.5	0.003	47	69.1	80	45.7	0.002
Other	127	49.0	47	38.2	69	57.5		21	30.9	95	54.3	
Unknown*	77	22.9	40	24.5	24	16.7		28	29.2	36	17.1	
**Pregnancy at Diagnosis** ^**2**^												
No	64	91.4	33	94.3	25	92.6	1	17	100.0	41	91.1	0.568
Yes	6	8.6	2	5.7	2	7.4		0	0.0	4	8.9	
Unknown*	10	12.5	6	14.6	3	10.0		2	10.5	7	13.5	
**Treatment Status** ^**2**^												
Naive	203	61.7	109	68.6	76	53.2	0.007	70	73.7	115	55.6	0.003
Treated	126	38.3	50	31.5	67	46.9		25	26.3	92	44.4	
Unknown*	7	2.1	4	2.5	1	0.7		1	1.0	4	1.9	
**Log**_**10**_ **Viral load [SD]** ^**3**^	4.4	[0.99]	4.4	[0.97]	4.4	[1.03]	0.988	4.5	[0.89]	4.3	[1.04]	0.131
**CD4 cell count** ^**4**^	441.5 (241–667)		493 (290–671)		356 (202–659)		0.046	553 (320–710)		372 (211–649)		0.01
**CD4 count categories** ^**1**^												
<200	64	20.5	26	17.5	33	24.3	0.025	13	14.6	46	23.5	0.013
200 − < 350	59	18.9	22	14.8	34	25.0		12	13.5	44	22.5	
350 − < 500	59	18.9	27	18.1	22	16.2		14	15.7	35	17.9	
500	130	41.7	74	49.7	47	34.6		50	56.2	71	36.2	
Unknown*	24	7.1	14	8.6	8	5.6		7	7.3	15	7.1	
**AIDS-defining illnesses** ^**2**^												
No	181	85.8	98	90.7	64	76.2	0.009	65	92.9	97	79.5	0.014
Yes	30	14.2	10	9.3	20	23.8		5	7.1	25	20.5	
Unknown*	125	37.2	55	33.7	60	41.7		26	27.1	89	42.2	
**CDC stage** ^**1**^												
1	67	33.7	47	46.1	15	19.2	0.0007	33	50.0	29	25.4	0.001
2	72	36.2	35	34.3	27	34.6		22	33.3	40	35.1	
3	60	30.2	20	19.6	36	46.2		11	16.7	45	39.5	
Unknown*	137	40.8	61	37.4	66	45.8		30	31.3	97	46.0	
**Recently infected** ^**2**^												
No	199	93.0	94	92.2	88	93.6	0.785	58	92.1	124	93.3	0.772
Yes	15	7.0	8	7.8	6	6.4		5	7.9	9	6.8	
Unknown*	122	36.3	61	37.4	50	34.7		33	34.4	78	37.0	
**HBsAg** ^**2**^												
Negative	310	95.1	154	96.9	128	92.1	0.077	93	97.9	189	93.1	0.103
Positive	16	4.9	5	3.1	11	7.9		2	2.1	14	6.9	
Unknown*	10	3.0	4	2.5	5	3.5		1	1.0	8	3.8	
**Anti-HCV** ^**2**^												
Negative	92	95.8	49	96.1	30	93.8	0.637	40	97.6	39	92.9	0.616
Positive	4	4.2	2	3.9	2	6.3		1	2.4	3	7.1	
Unknown*	240	71.4	112	68.7	112	77.8		55	57.3	169	80.1	
**HIV-1 subtype** ^**1**^												
A1	66	19.6	42	25.8	24	16.7	0.109	29	30.2	37	17.5	0.002
B**	183	54.5	95	58.3	86	59.7		60	62.5	121	57.4	
C	29	8.6	13	8.00	16	11.1		4	4.2	25	11.9	
F1	12	3.6	3	1.8	9	6.3		0	0	12	5.7	
CRF02_AG	19	5.7	10	6.1	9	6.3		3	3.2	16	7.6	
Others	27	8.1	NA		NA			NA		NA		

^*^For each variable, the total unknown values are shown, but these values were excluded from the sum of proportions and the statistical analysis.

^**^Two subtype B sequences were excluded from the final alignment.

^1^For categorical variables, associations were tested using Chi-squared test.

^2^For categorical variables, associations were tested using Fisher’s exact test.

^3^For continuous variables, associations were tested using t-test.

_4_For continuous variables, associations were tested using Mann-Whitney U test.

Demographic, epidemiological, behavioral and clinical data were collected by the medical and paramedical personnel of the AIDS Clinic of Larnaca General Hospital. Informed consent was obtained from each study subject, and data was double-coded to ensure patient anonymity. Recent infections were defined using a maximum of 6 months between the last seronegative and the first seropositive HIV-1 test^13^, a CD4 count >200 cells/ml and the absence of AIDS-defining conditions.

### Behavioral questionnaire: Risk factors at the time of infection

The risk factors included in the behavioral questionnaire concerned sexual behavior, including sexually transmitted disease (STD) diagnosis in the past two years, stable relationship with source, anonymous sex, and sex for money/drugs. Questions regarding the source of infection were also asked, such as knowledge of their identity, their seropositivity, their antiretroviral treatment and the scheme of combined antiretroviral treatment (cART). Unwillingness to answer these questions or the lack of knowledge was also included in the questionnaire. A detailed description of the epidemiological, behavioral and clinical data collected for the Cypriot patients was previously presented^4,5,11^.

### Subtyping and transmission cluster (TC) analyses

**Figure 1:**
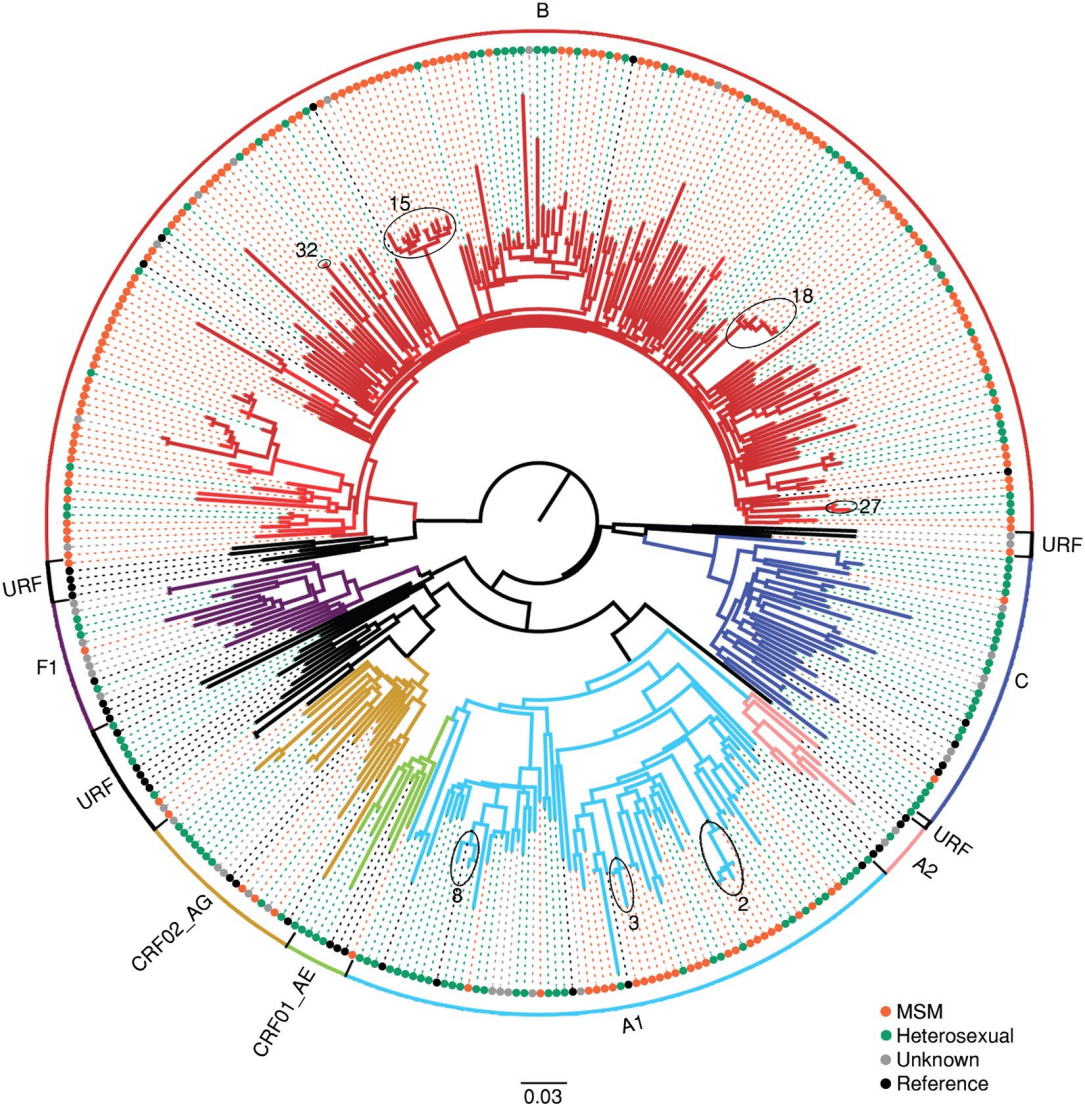
Phylogenetic trees. Maximum likelihood (ML) phylogenetic tree of the Cypriot cohort with the subtype references. The colored circles at the tips of the tree show the risk group and references of each subtype indicated by brackets. The phylogenetic clusters in subtypes A1 and B marked by ovals denote the presence of HIV-1 strains from Cypriot patients who were identified in transmission clusters in Cyprus in which two or more Cypriot patients are part of active transmission (see Methods section and Fig. 3). Phylogenetic clusters were defined by a subtype-specific Maximum Likelihood tree and using previously established criteria (bootstrap support greater than 70% and with a mean genetic distance of fewer than 0.045 nucleotide substitutions per site). The number associated to each phylogenetic cluster corresponds to the number of the corresponding transmission cluster in Fig. 3.

HIV-1 sequences encoding 1,106 nucleotides of the *pol* region (protease and partial reverse transcriptase) corresponding to positions 2253 to 3359 of the HXB2 genome (GenBank accession number K03455) were obtained from plasma from all participating study subjects^4,5,11^. Sequence contamination was routinely controlled using Neighbor Joining trees as part of sequence quality assurance for all newly obtained HIV-1 genomic sequences. All sequences forming clusters with low genetic distances can be explained by the epidemiological information collected from the study subjects (i.e. sexual partners). HIV-1 subtypes were determined with Rega (version 3)^14^ and COMET (version1.0)^15^. If assignment by the tools was discordant, traditional phylogenetic analyses were performed as previously described^14^. An initial tree containing the total cohort and subtype reference sequences retrieved from the Los Alamos database (www.hiv.lanl.gov) was generated using a maximum likelihood (ML) procedure with the GTR+4Γ model and 1000 bootstrap replicates in RAxML (version 8)^16^ (Fig. 1).

To determine the factors that are associated with the transmission of HIV, TC analyses were performed. For this analysis, only sequences classified as subtypes A1, B, C, F1, or CRF02_AG were included since they were predominant in this cohort. For the TC reconstruction, control sequences were added to this dataset. Control sequences were selected using the following procedure: (i) the 30 best-matched sequences to each sequence of the Cypriot cohort were retrieved by BLAST (http://blast.ncbi.nlm.nih.gov/Blast.cgi); (ii) the HIV-1 *pol* sequences from the SPREAD program, which collected data from newly diagnosed individuals in 26 countries between 2002 and 2010, were included in the analyses^17^; (iii) since there have been reports of epidemiological links between Greece and Cyprus^18^, all HIV-1 *pol* sequences available from Greece in the Los Alamos database (http://www.hiv.lanl.gov) were included along with sequences from a recent study performed in Northern Greece^18^; and (iv) three subtype D and B reference sequences were used as outgroups for the B and non-B subtypes, respectively (http://www.hiv.lanl.gov). Sequences with low quality according to the Calibrated Population Resistance Tool (http://cpr.stanford.edu), duplicates and clones were deleted. The resulting dataset was aligned by using the MUSCLE algorithm^19^ and edited with MEGA (version 6.0)^20^ separately for each subtype. To avoid potential bias caused by convergent evolution, the positions of identified mutations causing or contributing to HIV-1 drug resistance listed in the most recent surveillance of transmitted HIV-1 drug resistance (TDR) were removed from the alignment^21^. The final datasets contained 1,160 sequences (alignment length: 795 nucleotides) for subtype A1, 7, 194 sequences (alignment length: 795 nucleotides) for subtype B, 1, 170 sequences (alignment length: 795 nucleotides) for subtype C, 400 sequences (alignment length: 798 nucleotides) for subtype F1 and 720 sequences (alignment length: 798 nucleotides) for subtype CRF02_AG.

**Figure 2:**
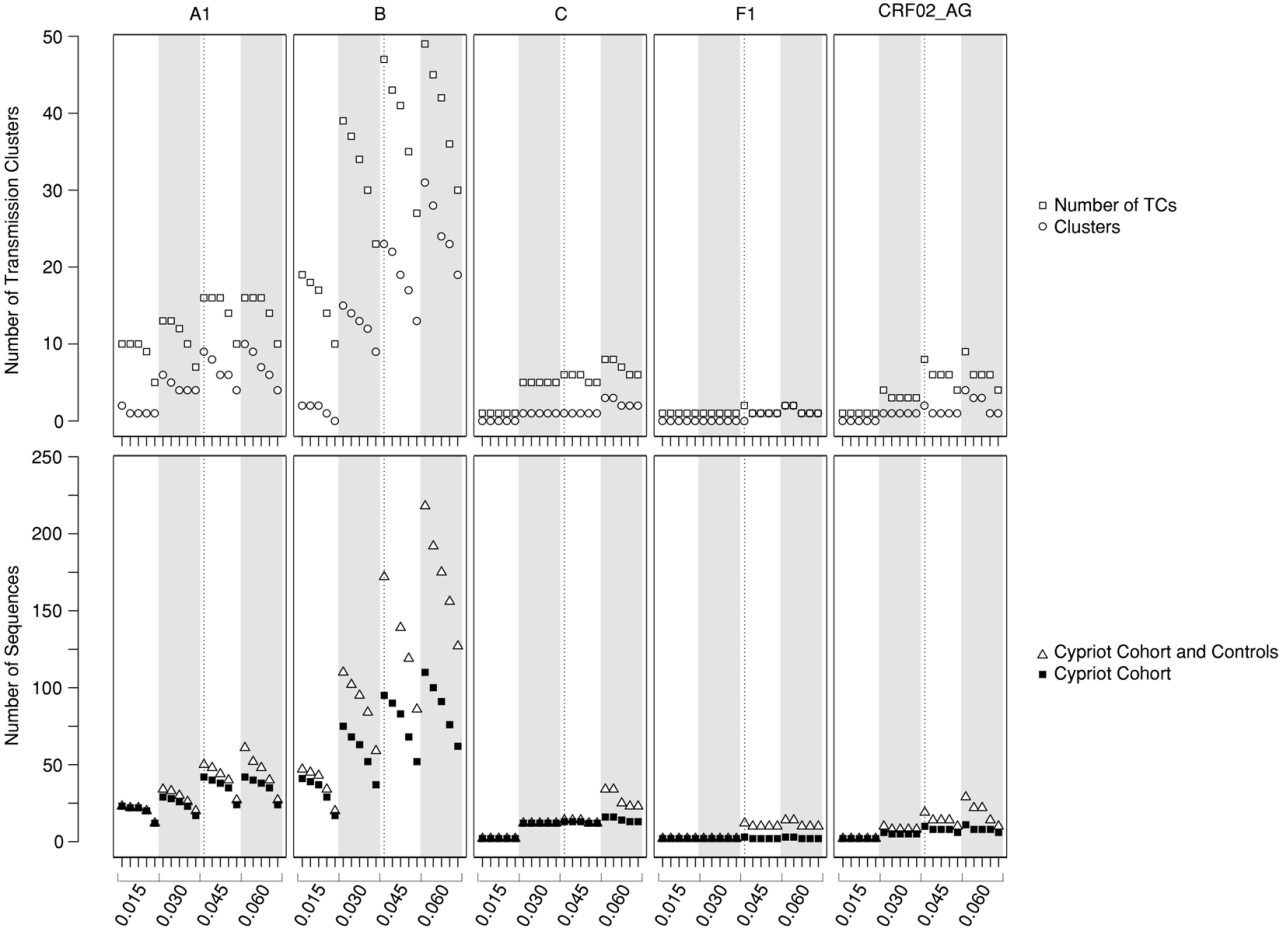
Sensitivity analysis. To evaluate the effect of the definition Transmission Cluster (TC) in the results, sensitivity analysis was performed for each combination of HIV-1 subtype (A1, B, C, F and CRF02_AG), genetic distance (0.015, 0.030, 0.045 and 0.060) and bootstrap threshold (70, 80, 90, 95, 98). The vertical dashed line is the threshold used in the analyses (genetic distance of 0.045 as the threshold and a bootstrap support of 70%). In the upper panel, the number of pairs are shown as open squares and clusters are shown as open circles. In the lower panel, the number of HIV-1 sequences from patients of the Cypriot cohort and the controls are shown as open triangles and the number of HIV-1 sequences from patients of the Cypriot cohort only are shown as closed squares. 1,094 control and 66 cohort sequences were analyzed for subtype A1; 7,011 control and 183 cohort sequences for subtype B; 1,141 control and 29 cohort sequences for subtype C; 388 control and 12 cohort sequences for subtype F1 and 701 control and 19 cohort sequences for subtype CRF02_AG.

An ML tree was generated for each subtype with the GTR+4Γ nucleotide substitution model using 1,000 bootstraps, implemented in RAxML (version 8.0)^16^. The TCs were identified with Cluster Picker^7^ using a genetic distance of 0.045 as the threshold and bootstrap support of 70%. To evaluate the effects of the definition of the TC in the results, a sensitivity analysis was performed and repeated for different genetic distances (0.015, 0.030, 0.045, and 0.060) and bootstrap support values (70, 80, 90, 95, and 98). In total, 20 different combinations of genetic distance and bootstrap support values were tested per subtype (Fig. 2). The TCs were classified as pairs or as clusters (with ≥3) of patients since the clusters with three or more individuals suggested onward transmission of HIV-1.

### Bayesian phylogenetic analyses

To confirm the TCs and calculate the most recent common ancestor (MRCA)^9^, a Bayesian Monte Carlo-Markov Chain (MCMC) inference was performed using the program BEAST (version 1.8.2)^22^. The TCs were grouped together with additional controls for the known year of sampling. The temporal signal was evaluated by using the cross-platform algorithm, TempEst (TEMPoral Exploration of Sequences and Trees, formerly known as Path-O-Gen)^23^. The uncorrelated log-normal relaxed molecular clock with a discretized GTR substitution model was used, as was the Bayesian Skygrid coalescent model with 50 grid points and a cutoff value specific to the MRCA of each subtype^24–26^. Analyses were run in triplicate for at least 100 million MCMC generations. Convergence was evaluated using Tracer with a burn-in of 10% (http://beast.bio.ed.ac.uk/Tracer). The maximum clade credibility (MCC) tree was summarized with TreeAnnotator after discarding the burn-in and visualized with FigTree (version 1.4.2) (http://tree.bio.ed.ac.uk). To evaluate the active TCs or the onward transmission of HIV-1 in recent years, a sub-analysis was performed using a cluster depth of less than or equal to five years^27^, corresponding to the period between 2008 and 2012 for this study. This depth was calculated as the length of time between the ancestral node and the most recent tip of the tree. Therefore, a TC was potentially divided into two or more sub-clusters.

### Statistical analyses

For continuous variables, comparisons between groups were conducted using a t-test or the Mann-Whitney U test. For categorical variables, comparisons between proportions were conducted using the contingency-table Chi-squared test, Fisher’s exact test or regression techniques as appropriate. A binomial logistic regression was performed to determine the factors that were associated with clustering. All analyses were performed for the TCs (pairs and clusters) and were then repeated only for clusters. The level of statistical significance was set at 5%. A linear trend in proportions across sampling years was assessed using the Cochran-Armitage test. Data analyses were performed with the statistical software R v.3.3.1.

## Results

### The Cypriot cohort primarily consists of young Cypriot males who reported men who have sex with men (MSM) as a mode of HIV-1 transmission

In total, 336 study patients were included in the final analysis. Importantly, the number of study subjects represents 84.8% (336/396) of HIV-1-infected people linked to care between 1986 and 2013^2^. However, this number accounts for only 42.3% (336/794) of the total cumulative cases reported by the ECDC between 1986 and 2012^12^. To determine whether this cohort is representative of the total cumulative cases reported in Cyprus, data from our cohort (Table 1) were compared with the available data collected from the ECDC^2,12^. The cumulative percentage of MSM was 36% (compared to 52.1% in the Cypriot cohort, p < 0.0001), the cumulative percentage of heterosexuals was 57.3% (compared to 41.0% in the Cypriot cohort, p < 0.0001), the cumulative percentage of intravenous drug users (IVDU) was 1.1%, and the cumulative percentage of those with vertical transmission was 0.4%^25^. People with acquired immune deficiency syndrome (AIDS) at the time of diagnosis comprised 30.6% (p > 0.05).

Most patients in the cohort were males between 25 and 34 years of age who originated from Cyprus and reported MSM as the mode of transmission. These patients were infected with subtypes B or A1 and were treatment-naïve at the time of diagnosis (Table 1, Fig. 1). Heterosexuals and MSM together accounted for 87.2% (293/336) of the study patients. The mean age of the MSM group was 36.9 years (standard deviation (SD): 9.8) compared to 40.9 years (SD: 13.3) for the heterosexual group (p = 0.005). In total, 89% of the MSM group originated from Cyprus, and 42.6% of the heterosexual group originated from abroad, mainly from Eastern Europe (8.2%), Middle Africa (9.3%) and Western Asia (5.4%). The MSM group was mostly infected with subtype B (76.2%), while the heterosexual group was mostly infected with non-B subtype viruses (62.8%) (p < 0.001) (Fig. 1). Cypriots reported MSM as the main mode of transmission (62.6%). Non-Cypriots reported being heterosexual (53.4%). In total, 67.4% of Cypriots were infected with subtype B, while only 25.2% of non-Cypriots were infected with subtype B (odds ratio (OR): 6.08, confidence interval (CI): 3.5–10.7; p < 0.0001). In a multivariate analysis, Cypriots were more likely to be male (OR: 3.0, 1.4–6.6), older (OR: 1.9, 1.05–1.12), MSM (OR: 3.8, 1.7–8.7) and infected with subtype B (OR: 3.1, 1.7–5.9) than non-Cypriots. The number of newly diagnosed patients included in the Cypriot cohort (n = 203) that originated abroad (n = 70) did not increase over time (p = 0.59).

### Anonymous sex is perceived by MSM as an important risk factor for contracting HIV-1, but it is not a determining factor for the onward transmission of HIV-1 in Cyprus

Among newly diagnosed patients (n = 203), 48.8% reported anonymous sex as a relevant risk factor at the time of infection, but this trend has decreased since 2009 (p = 0.0003). Anonymous sex was more frequently reported by men (55.5%; OR: 8.4, 4.4–17.0; p < 0.0001) and people who originated from Cyprus (47.8%; OR: 2.5, 1.5–4.3; p = 0.0004). Seventy-six percent of MSM reported anonymous sex (114/150; OR: 3.4, 2.0–5.8; p < 0.0001) at the time of infection compared with 53.3% (65/122) of heterosexuals. In a multivariate model, MSM was the only variable that remained associated with reporting anonymous sex as a risk factor at the time of infection (OR 3.4 CI: 1.9–6.0; p < 0.0001). However, the proportion of MSM who reported anonymous sex was similar to that of individuals who were or were not included in the identified TCs (32% in each group). Only approximately 4% (15/336) of the total cohort reported having been diagnosed with an STD in the past two years; however, the proportions of these patients were similar for individuals who were or were not included in the identified TCs (50% in each group). Women (62.0%) and heterosexuals (43.8%) were more likely to know who the source of HIV infection was (OR for females: 6.5, 3.5–12.5; OR for heterosexuals: 2.9, 1.7–5.0; p < 0.0001), while only 20.9% of the MSM population was aware of the source. However, only 3% (9/301) of the respondents, primarily heterosexual women and those in a stable relationship (n = 7), were aware that the source of the infection was positive. Five of these women and two of the men in a stable relationship (2%) knew that the source had taken ART.

### Younger patients who reported being infected in Cyprus are associated with the onward transmission of HIV-1

**Figure 3:**
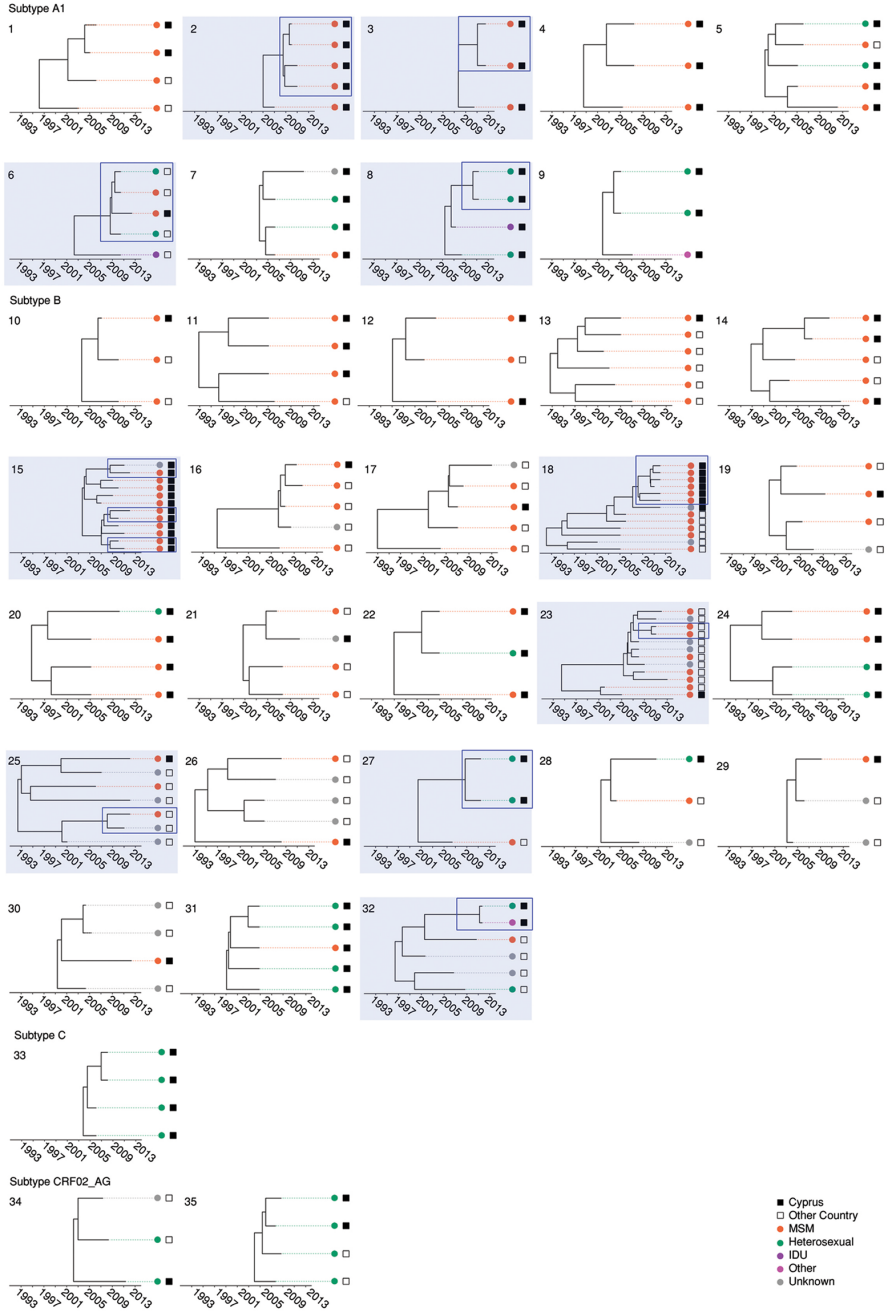
Transmission Clusters (TC). Thirty-five clusters were identified. Clusters 1–9 belong to subtype A1, clusters 10–32 to subtype B, cluster 33 to subtype C and clusters 34–35 to subtype CRF02_AG. The TCs are numbered according to HIV-1 subtype (A1, B, C, F and CRF02_AG) and the decreasing percentage of men having sex with men (MSM) individuals in the clusters within each subtype. TCs that contain active clusters are highlighted in light blue and clusters that were still active in the past five years according to the depth of the cluster are outlined in dark blue rectangles. The colored circles at the tips of the tree show the risk group and squares represent the country of sampling (see color code at the bottom of the figure).

In total, 79 TCs were identified, including 163 (53.1%) Cypriot patients and 96 controls. The median number of patients in the TCs was 2 (IQR: 2–4). Thirty-five TCs were clusters, including 96 patients from Cyprus and 75 reference patients (Fig. 3). The characteristics of the patients within or outside of the TCs are shown in Table 1. Regarding risks at the time of infection, 55.2% of the population in TCs reported anonymous sex, and 35.6% of the population knew who infected him/her. In the multivariate analysis, being infected in Cyprus (OR: 2.21, 1.26–3.92, p = 0.013) and being drug-naïve (OR: 2.18, 1.24–3.86, p = 0.009) were identified as independent predictors of the clustering of pairs and clusters, while age (OR: 0.54 for each 10-year increment; CI: 0.36–0.77, p = 0.002) and being infected in Cyprus (OR: 3.14, 1.55–6.65, p = 0.031) were predictors of clustering with ≥3 patients.

To verify the reliability of our findings, a sensitivity analysis was performed to evaluate how the threshold influenced the results (Fig. 2). Despite varying numbers of TCs and patients, age and country of infection were consistently predictors of clustering, even when the most stringent cutoff of 0.015 and a 95% bootstrap value were used. Regarding the MRCA of the identified TCs, we identified 29 Cypriot patients (29/163; 18%) belonging to 14 TCs (14/79) who were registered at the AIDS Clinic before the introduction of cART in Cyprus in 1996. The remaining Cypriot patients (134/163) were involved in the TCs with an estimated MRCA between 1996 and 1999 (24%), 2000–2004 (37%) or 2005–2012 (21%). The 96 Cypriot patients belonging to TCs with ≥3 patients were involved in TCs with the following estimated MRCAs: before 1996, 26%; 1996–1999, 27%; 2000–2004, 34%; and 2005–2012, 13%.

### Two distinct sub-epidemics of young MSM and heterosexuals who reported being infected in Cyprus are driving the transmission of the subtype A1 sub-epidemic in Cyprus

**Figure 4:**
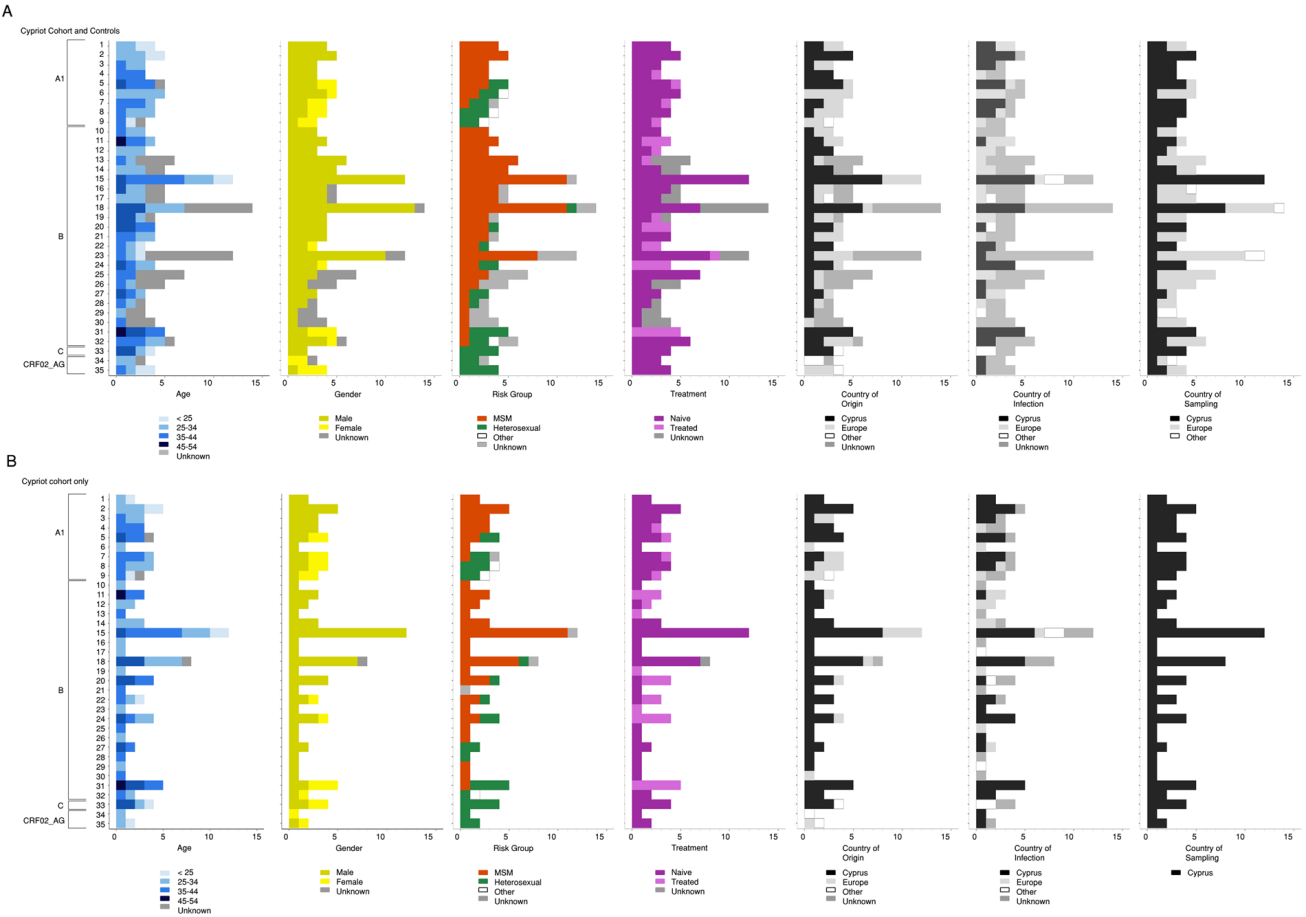
Epidemiological and demographic information of transmission clusters. For each TC, epidemiological (age, gender, risk group and treatment) and demographic (country of origin, country of infection and country of sampling) are illustrated. **(A)** Cypriot cohort and controls and **(B)** Cypriot cohort alone.

The subtype A1 sub-epidemic included seven pairs and nine TCs with ≥3 patients (numbers 1 to 9 in Figure 3); 63.6% (42/66) of the Cypriot population was involved in the TCs, and eight controls were sampled, primarily from Greece (n = 7). The median size of the TCs was 3 (IQR: 2–4). The population in the A1 TCs largely consisted of males (28/42, 66.7%) aged between 25 and 44 years (70.0%) who originated from Cyprus (59.5%), resided in Nicosia (45.2%) and were treatment-naïve (78.6%). Anonymous sex was reported as a risk factor at the time of infection by 35.7% of cases, followed by stable relationships (33.3%) and being treatment-naïve (78.6%). Interestingly, two clearly distinct sub-epidemics were identified comprising similar numbers of MSM and heterosexuals in the TCs (45.2% and 42.8%, respectively). For example, the MSM-driven epidemic included five clusters with a median size of 4 and a total of 20 individuals who originated from Cyprus (n = 15) and reported that they were infected in Cyprus (n = 11). The heterosexual epidemic, however, included 3 clusters with a median size of 3 and a total of 11 individuals who were female (n = 6) and originated from different regions in Europe (n = 8) but had limited information regarding the possible country of infection (Cypriots n = 5, European = 2, remaining unknown). In the univariate analysis of clusters, the patients in the clusters were significantly younger (31.3 SD: 9.6 versus 39.8 SD: 12.3) and were more likely to have originated from Larnaca (34.5% versus 8.1%), to have been infected in Cyprus (80.0% versus 50.0%), to be MSM (60.7% versus 26.5%) and to be treatment-naïve (86.0% versus 59.0%). In the multivariate analysis, being younger (OR: 0.36 for every 10-year increment; CI: 0.14–0.76), being MSM (OR: 9.7, 1.6–70) and becoming infected in Cyprus (OR: 10.8, 1.7–118) were significantly associated with clustering. Age and Cyprus as the likely country of infection were consistently significant in the sensitivity analyses, with the exception of risk factor analysis. Subsequently, clusters containing primarily MSM were compared with the remaining clusters (Fig. 4). The country of origin and the city of residence were significant in the univariate analysis, and being Cypriot was significantly associated with MSM clusters (OR: 11.8, 2.12–93.1; p = 0.002).

Considering the last five years of the study, five active clusters were identified for subtype A1, including three pairs and two clusters of four patients who each resided within TCs (see Fig. 3). Of the 14 patients in these clusters, 57.1% were MSM, 28.5% originated from Cyprus and 50.0% were infected in Cyprus. Overall, these results support the existence of two distinct sub-epidemics driving the spread of subtype A1. The first cluster (number 2 in Fig. 3) included four treatment-naïve MSMs from the Cypriot cohort who originated from and were infected in Cyprus. The second cluster (number 3 in Fig. 3) included two treatment-naïve MSMs from the Cypriot cohort who originated from Bulgaria but were infected in Cyprus. The third cluster (number 6 in Fig. 3) included four treatment-naïve individuals, of which one was an MSM from the Cypriot cohort who originated from and was infected in Greece. The three controls were one MSM and a heterosexual male who originated from and were infected in Greece and a heterosexual female who originated from Russia. The fourth cluster (number 8 in Fig. 3) included a heterosexual male and a female from the Cypriot cohort. These individuals were treatment-naïve and originated from Greece and Romania, respectively, but both were infected in Cyprus. The fifth active cluster (not shown) represented a TC pair that included a male and female from the Cypriot cohort with no information on the risk group. Both subjects were treatment-naïve and originated from Ukraine. The male individual was infected in Cyprus, while no information was available for the female individual. Overall, these results suggest that the active subtype A1 TCs are primarily driven by MSM who originated from Cyprus or Greece.

### Young patients drive the transmission of the subtype B sub-epidemic in Cyprus

**Figure 5:**
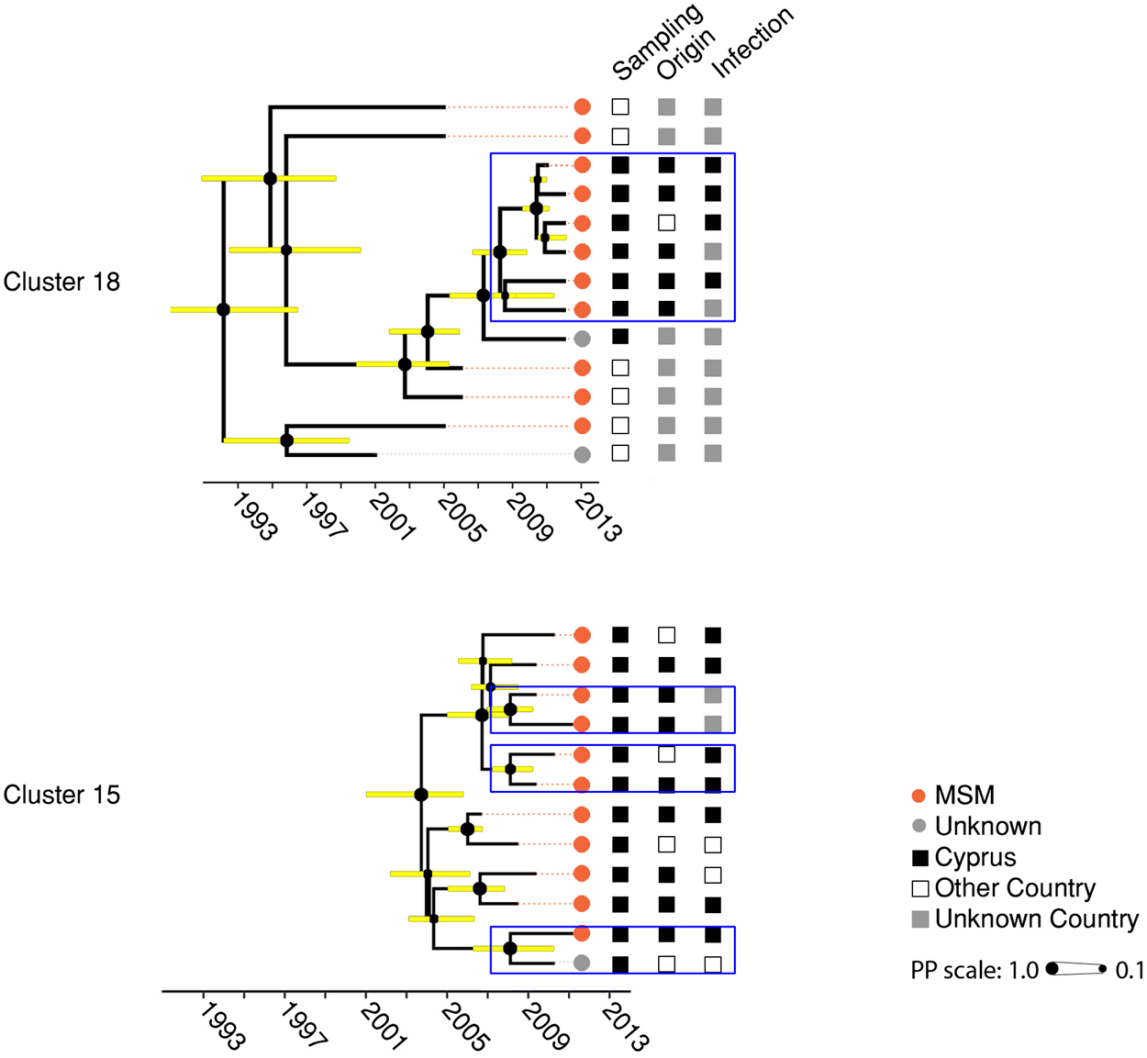
Subtype B MSM transmission clusters (TCs). The two largest subtype B TCs (#15 and #18, respectively) with mainly Cypriots who reported being MSM are illustrated with additional demographic information. The upper TC (#18) originated in 1992 [Bayesian Credible Interval (BCI): 1987–1996], while the lower TC originated in 2003 [2004–2006]. Horizontal bars indicate the BCI of each node. The dashed gray line indicates the past five years or the cluster depth. The colored circles at the tips of the tree show the risk group and squares represent the country of sampling, the country of origin and the country of infection, respectively. Active clusters within the TCs are outlined in dark blue rectangles.

Twenty-four pairs and 23 clusters were identified for subtype B (numbers 10 to 32 in Fig. 3). This sub-epidemic included 52.4% (95/181) of individuals in TCs from the Cypriot cohort and 77 controls. The median size of the TCs was 2 (IQR: 2–4). Individuals in the TCs were primarily males (86.3%) between 25 and 44 years old (67.4%) who originated from Cyprus (83.2%), mostly resided in Limassol (41.0%) and Nicosia (33.7%), were mostly MSM (68.4%), were treatment-naïve (58.9%), reported anonymous sex (57.9%) as a relevant risk factor at the time of infection, and were treatment-naïve (58.9%). The patients in two TCs are shown in more detail in Fig. 5. Cluster 15 included twelve individuals who were primarily MSM sampled in Cyprus and originated from Cyprus (n = 8), Greece (n = 2), the United Kingdom (n = 1) and Serbia (n = 1). Cluster 18 included fourteen patients, who were also largely MSM (n = 11). Eight patients were sampled in Cyprus and the remaining in the United Kingdom (n = 5) and Australia (n = 1). Seven patients originated from Cyprus and one from Greece; five reported Cyprus as the country of infection.

Fourteen clusters with a majority of MSM patients had a median size of 4.5 and a total of 84 patients. These clusters also included other Europeans who originated from Greece (n = 6), Poland (n = 3), Germany (n = 3,), United Kingdom (n = 2), Belgium (n = 2), Austria (n = 2), other European countries (n = 6) and Brazil (n = 1). If the country of sampling was considered, most individuals were sampled in the United Kingdom (n = 15), Poland (n = 7), Greece (n = 5), Austria (n = 2), Belgium (n = 2), Germany (n = 2), other European countries (n = 6) and Hong Kong (n = 1). Thirty-four individuals reported the country of infection, and 47% of the infections occurred in Cyprus, 18% of the infections occurred in Greece, and the remaining infections occurred in other European countries. Conversely, only two clusters primarily containing heterosexuals were identified, including eight individuals who largely originated from and reported being infected in Cyprus (Fig. 4).

When TCs were considered, only the CD4 count and CDC stage were significant in the univariate analysis. In the multivariate analysis, no variables were significant. When considering only clusters, age was significantly associated with clustering in the univariate and multivariate analyses (OR: 0.6 for every 10-year increment; CI: 0.42–0.95). This result was consistent in the sensitivity analysis, even when a threshold of 0.015 and a 98% bootstrap value were used. In a sub-analysis to evaluate the differences in MSM majority clusters versus clusters with a majority of other risk factors, no significant differences were observed (Fig. 4).

Considering the last five years of the study, eight active clusters were identified for subtype B, with seven pairs and one cluster of 7 individuals all within the TCs shown in Fig. 3. Overall, all individuals in the active TCs were treatment-naïve, 66.6% (14/21) were MSM, 66.6% (14/21) originated from Cyprus and 52.3% (11/21) were infected in Cyprus. These results suggest that active clusters of subtype B may be driven by young, drug-naïve individuals who are primarily MSMs.

The fourth active cluster (number 18 in Figs 3 and 4) included 7 treatment-naïve individuals from the Cypriot cohort, of which six were MSM, and one was a heterosexual male. Six individuals originated from Cyprus and one from Greece, and five were infected in Cyprus. The fifth cluster (number 23 in Fig. 3) included two who were MSM from the control patients, with no further information available. The sixth cluster (number 25 in Fig. 3) included two treatment-naïve controls, and the only available information indicated that one individual was MSM. The seventh cluster (number 27 in Fig. 3) presented two treatment-naïve heterosexual males from the Cypriot cohort. Both originated from Cyprus and were infected in Greece and Cyprus. Both individuals in the eighth cluster (number 32 in Fig. 3) were treatment-naïve and from the Cypriot cohort. A heterosexual female and a female from the unknown risk group originated from and were infected in Cyprus. Overall, all 21 individuals in the active TCs were treatment-naïve, 14 were MSM, and 14 originated from Cyprus. These results suggest that the active clusters of subtype B may be driven by young, treatment-naïve individuals who are primarily MSM.

### Limited transmission of other subtypes (not A1 and B) in Cyprus occurs primarily among heterosexuals

The TCs were limited for the other subtypes. For subtype F, only 2 pairs were identified, representing 25% of the cohort infected with subtype F strains. For subtype C, 5 pairs and 1 cluster with ≥3 patients (number 33 in Fig. 3) were identified, which included 44.8% of the cohort infected with subtype C strains. For subtype CRF02_AG, 6 pairs and 2 clusters (numbers 34 and 35 in Fig. 3) were identified and collectively accounted for 52.6% of the patients infected with CRF02_AG strains. Heterosexuals were the main risk group included in these TCs (57.9%). There was only one active pair for subtype F, consisting of two patients sampled in Cyprus but with unknown socio-demographic data.

### The proportion of females and heterosexuals involved in clusters is higher in the Cypriot cohort than in other European countries

To determine whether the patterns of transmission in the Cypriot epidemic are different from those in the European epidemic, TCs with at least one individual sampled in Europe and no patients from the Cypriot cohort were retrieved. The median size of the European clusters was 4 (IQR: 3–5), including 1,498 individuals in 749 pairs and 2,232 in 483 clusters. Based on the population in the TCs with available data, the mean age was 35.4 years (SD: 9.7), 89.9% were male (3,089/3,437), 78.9% (2,352/2,982) were MSM, 21% were heterosexuals, and 98.7% (3,544/3,591) were naive. When considering patients in TCs or clusters in the European versus Cypriot cohorts, the Cypriot cohort had a higher proportion of females and heterosexuals (p < 0.0001). However, this difference was no longer significant when the subtype A1 and B sub-epidemics were considered independently (Fig. 4).

## Discussion

This study used in-depth statistical and phylogenetic analyses to analyze the HIV-1 epidemic in Cyprus. We analyzed a cohort in which 85% of all patients were linked to care, and we used socio-demographic, clinical, risk factor, and virological data from the time of infection to understand how HIV-1 transmission is occurring in the country. This effort involved an extensive sampling of HIV-1 patients. However, the percentage of patients linked to care in Cyprus is low due to emigration from the country^2^. Thus, our cohort accounts for 42.3% (336/794) of the total cumulative reported cases between 1986 and 2012^12^. Because we have a higher proportion of MSM compared to other risk factors than the proportions reported by the ECDC^12^, some bias may be associated with this analysis. However, we attempted to decrease the bias by using different databases from Greece and Europe with complete socio-demographic data to identify and include patients who originated from Cyprus but were sampled elsewhere. Another source of caution is the lack of a consensus regarding the definition of TCs and the intrinsic limitations of the phylogenetic analyses^28^. We attempted to decrease the effects of these limitations by including a dense Cypriot sample and several controls from other countries and conducting a sensitivity analysis to evaluate the robustness of our findings. Notably, most of our extrapolations of the parameters associated with the TCs were robust, using different thresholds. Therefore, the definition of a threshold used for the analysis did not influence the main findings described in this manuscript.

We found two distinct and concurrent HIV-1 sub-epidemics circulating in the country, i.e., subtypes A1 and B, and both sub-epidemics are driven by young people. Importantly, the A1 sub-epidemic is primarily driven by MSM, followed by heterosexuals. This finding is different from those from other European cohorts, in which MSM are usually only associated with the transmission of subtype B infections^29,30^. Furthermore, the MSM populations involved in these two sub-epidemics primarily originated from and were infected in Cyprus and were significantly younger and more likely to have engaged in anonymous sex at the time of infection than the heterosexual population. Additionally, the active HIV-1 clusters, i.e., those in which transmission occurred most recently, were also driven by MSM who originated from and were infected in Cyprus. This finding suggests that prevention policies should target the young MSM population originating from and living in this country.

One gap in the surveillance of the HIV epidemic in Cyprus is the cause for the increase in the number of new diagnoses in the past several years^2^. A hypothesis has been that the increase in new diagnoses may be attributable to the increased number of migrants in the country. However, our results indicate that a) new diagnoses of HIV-1 in migrants have been constant, which is consistent with the ECDC report^2^, and b) migrants who are infected with HIV-1 are not predominantly or actively transmitting the overall epidemic.

The clustering analyses provided further insights regarding the relationship between this epidemic and those in other European countries because these results were not obtained by performing classical epidemiological studies. We found that one-third of the clusters with ≥3 patients were domestic, and two-thirds showed evidence of origin/infection/sampling in other European countries (Fig. 4). If the subtype is also considered, two-thirds of the patients in the subtype B clusters originated or were infected/sampled in Europe, compared to only half of the patients in subtype A1. This finding indicates a higher level of connectivity of the subtype B sub-epidemic with other countries than the subtype A1 sub-epidemic, which is not surprising considering the older origin of the subtype B epidemic. However, these findings also suggest that the increase in new infections is explained by sexual tourism in and out of the country. More studies will be needed to evaluate the directionality of these links. A high prevalence of subtype A1 has not been reported in European countries, with the exception of Cyprus^4,5,10,11^, Greece^18,29,31,32^ and Albania^33^. Classical epidemiology suggests that the subtype A1 sub-epidemic in Cyprus is affecting MSM who originated from both Cyprus and Greece and heterosexuals who originated primarily from Eastern Europe. However, our TC analysis allows us to further extrapolate that the patients who are involved in the transmission of subtype A1 are largely MSM who report being infected in Cyprus. More research is needed to clarify the relationship of this epidemic with countries other than Greece.

To the best of our knowledge, no other studies using similar methodology have analyzed sex behavioral factors at the time of infection as a determinant in the transmission of HIV as analyzed in TCs. This may be due to the small size of the population in Cyprus, which is approximately one million people, and the presence of only one AIDS clinic in the country, which facilitates the establishment of a densely sampled cohort study representing HIV-1-infected individuals in Cyprus and the collection of detailed clinical, epidemiological and behavioral information from study subjects. In this cohort, anonymous sex was reported in half of cases, and the likelihood of MSM being engaged in anonymous sex was three times greater. This finding may indicate a role for this behavioral pattern in the transmission of HIV-1 infection. However, based on the TC analysis, the proportion of MSM who reported anonymous sex and were present in clusters was similar to that of MSM who reported anonymous sex and were outside the clusters, suggesting that anonymous sex is not a determining factor in the onward transmission of the virus. Other behavioral factors may, therefore, play a role in the transmission of HIV, such as the use of condoms, sero-sorting and sex abroad. Despite the lack of an association between the onward transmission of HIV and the behavioral factors that were analyzed, our results highlight the importance of education regarding frequent testing for STDs and prevention strategies that target the MSM population.

In conclusion, using a representative sample from Cyprus we have shown two concurrent sub-epidemics, i.e., subtypes A1 and B, that are driven by young people. Our study clearly shows that phylogenetic and TC analyses are highly useful for gaining an in-depth understanding of the epidemic spread of HIV-1. Furthermore, our study sheds light on the possible causes of the increase in the number of new infections in Cyprus, which highlights the importance of targeting prevention policies toward young people who are infected in the country.
